# Downregulation of long non-coding RNA nuclear enriched abundant transcript 1 promotes cell proliferation and inhibits cell apoptosis by targeting miR-193a in myocardial ischemia/reperfusion injury

**DOI:** 10.1186/s12872-019-1122-3

**Published:** 2019-08-07

**Authors:** Lingyun Ren, Shanshan Chen, Wei Liu, Pan Hou, Wei Sun, Hong Yan

**Affiliations:** 1 Department of Anesthesiology, The Central Hospital of Wuhan, Tongji Medical College, Huazhong University of Science and Technology, 26, Shengli Street, Jiang’an District, Wuhan, 430014 China; 2 Key Laboratory for Molecular Diagnosis of Hubei Province, The Central Hospital of Wuhan, Tongji Medical College, Huazhong University of Science and Technology, 26, Shengli Street, Jiang’an District, Wuhan, 430014 China

**Keywords:** Long non-coding RNA nuclear enriched abundant transcript 1, Myocardial ischemia/reperfusion injury, miR-193a, Cell proliferation, Cell apoptosis

## Abstract

**Background:**

This study aimed to investigate the effect of long non-coding RNA nuclear enriched abundant transcript 1 (lnc-NEAT1) on cell proliferation and apoptosis in myocardial ischemia/reperfusion (I/R) injury cells, and explore its target miRNAs.

**Methods:**

H9c2 cells were cultured in oxygen and glucose deprivation followed by reperfusion (OGD/R) condition to construct a myocardial I/R injury model. Blank shRNA and lnc-NEAT1 shRNA were transferred into normal H9c2 cells and I/R injury H9c2 cells as Normal&sh-NC, OGD/R&sh-NC and OGD/R&sh-NEAT1 groups. Rescue experiment was performed by transfection of NC inhibitor plasmids, miR-193a inhibitor plasmids and NEAT1 shRNA into I/R injury cardiocytes. RNA expression, cell proliferation and cell apoptosis rate were detected by qPCR, CCK-8 and AV/PI respectively.

**Results:**

After OGD/R induction, H9c2 cell apoptosis was greatly increased while cell proliferation was decreased, which indicated successful establishment of myocardial I/R injury model, and lnc-NEAT1 expression was elevated as well. Cell proliferation rate was increased in OGD/R&sh-NEAT1 group at 48 h and 72 h compared to OGD/R&sh-NC group, while cell apoptosis was reduced in OGC/R&sh-NEAT1 group compared to OGD/R&sh-NC group. Target miRNAs detection indicated the negative regulation of lnc-NEAT1 on miR-193a but not miR-182 or miR-141. In rescue experiment, downregulation of lnc-NEAT1 promoted cell proliferation and inhibited cell apoptosis through targeting miR-193a in I/R injury H9c2 cells.

**Conclusion:**

Lnc-NEAT1 is overexpressed in myocardial I/R injury cells compared to normal myocardial cells, and downregulation of lnc-NEAT1 enhances cell proliferation while inhibits cell apoptosis through targeting miR-193a in I/R injury H9c2 cells.

## Background

Cardiovascular disease, a class of diseases involving the heart or blood vessels, has been reported to be the leading cause of deaths worldwide, which is responsible for approximately 17.3 million deaths in 2013 around the world, and increases rate of deaths from 25 to 40% during the period from 1990 to 2010 in China [1–3]. Most of the cardiovascular disease patients would generate myocardial ischemia when blood flow to heart is decreased due to a partial or complete blockage of cardiovascular arteries, which reduces the acquisition of enough oxygen, damages heart muscle and causes serious abnormal heart rhythms [4]. For settling this problem, reperfusion is popularly utilized, which delivers oxygen and nutrients to cardiac myocytes, subsquently sustaining aerobic metabolism and ATP generation, while it may cause paradoxical cardiomyocyte dysfunction as well as result in tissue damage [5]. These additional damages are defined as myocardial ischemia/reperfusion (I/R) injury, which leads to worse cardiovascular outcomes, suc h as stroke, myocardial infarction or even sudden death [5–7]. Thus, exploring novel mechanisms of myocardial I/R injury is of great importance for its prevention and treatment.

Long non-coding RNAs (lncRNAs), a type of RNAs with over 200 nucleotides in length and characterized by no protein coding properties, emerge as crucial mediators in several biological processes (such as cell proliferation, cells differentiation as well as apoptosis), among which parts of these lncRNAs appear with dysregulated and devote into the development and progression of various diseases (particularly cardiovascular diseases) [8–11]. LncRNA nuclear enriched abundant transcript 1 (lnc-NEAT1) is transcribed by RNA polymerase II from a common promoter and widely expressed in mammalian cells, which functions as scaffolds of nuclear bodies [12–14]. Accumulating evidences have revealed that lnc-NEAT1 regulates cells activities through targeting multiple miRNAs or signaling pathways in several diseases including traumatic brain injury, diabetes mellitus as well as carcinomas [15–17]. Considering information about the underlying mechanism of lnc-NEAT1 in myocardial I/R injury is rarely known, we conducted this study to investigate the effect of lnc-NEAT1 on cell proliferation and apoptosis in myocardial I/R injury cells, and explore its target miRNAs.

## Methods

### Cells culture

Rat cardiac muscle cell line H9c2 was purchased from Cell Resource Center of Shanghai Institute of Life Sciences, Chinese Academy of Sciences (Shanghai, China), and H9c2 cells were cultured in 90% Dulbecco’s Modified Eagle’s Medium (DMEM) medium (Gibco, USA) complimented with 10% Fetal Bovine Serum (FBS) (Gibco, USA). Cells were cultured in an incubator with 95% air and 5% CO_2_.at 37 °C.

### Construction of myocardial I/R injury model

H9c2 cells were cultured in oxygen and glucose deprivation followed by reperfusion (OGD/R) condition to construct a myocardial I/R injury model, and the detailed process was as follows: (1) H9c2 cells were firstly cultured in glucose-free DMEM (Gibco, USA) medium and incubated in oxygen-free atmosphere (95% N_2_ and 5% CO_2_ at 37 °C) for 10 h; (2) cells were then cultured in normal DMEM (Gibco, USA) medium under normal atmosphere (95% air and 5% CO_2_.at 37 °C) for another 24 h; (3) AV/PI was used to determine cell apoptosis rate to confirm the construction of myocardial I/R injury model; (4) qPCR was used to determine lnc-NEAT1 expression.

### Effect of lnc-NEAT1 shRNA on cell proliferation and apoptosis in myocardial I/R injury model

Blank shRNA and lnc-NEAT1 shRNA plasmids were constructed by Shanghai QeeJen Bio-tech Institution (Shanghai, China), and then transferred into normal H9c2 cells and I/R injury H9c2 cells using HilyMax (Dojindo, Japan). Cells were divided into 3 groups according to different interventions as follows: (1) Normal&sh-NC group (blank shRNA transferred into normal H9c2 cells); (2) OGD/R&sh-NC group (blank shRNA transferred into I/R injury H9c2 cells); (3) OGD/R&sh-NEAT1 group (lnc-NEAT1 shRNA transferred into I/R injury H9c2 cells). Subsequently, lnc-NEAT1 expression was detected by qPCR at 24 h, cell proliferation was detected by CCK-8 at 0 h, 24 h, 48 h and 72 h, cell apoptosis rate was detected by AV/PI at 72 h.

### Prediction and measurement of target miRNAs of lnc-NEAT1

Target miRNAs of lnc-NEAT1 in I/R injury cardiocytes was predicted using starBase and miRcode database, and three miRNAs related to I/R injury were selected to be measured by qPCR in this study: miR-141, miR-182 and miR-193a.

### Effect of Lnc-NEAT1/miR-193a on cell proliferation and apoptosis in myocardial I/R injury model

I/R injury cardiocytes were then transferred by (1) NC inhibitor plasmids (NC-inhibitor group); (2) miR-193a inhibitor plasmids (miR-inhibitor group); (3) NEAT1 shRNA and NC inhibitor plasmids (sh-NEAT1&NC-inhibitor group); (4) NEAT1 shRNA and miR-193a inhibitor plasmids (sh-NEAT1&miR-inhibitor group). Subsequently, lnc-NEAT1 expression was detected by qPCR at 24 h, cell proliferation was detected by CCK-8 at 0 h, 24 h, 48 h and 72 h, cell apoptosis rate was detected by AV/PI at 72 h.

### qPCR

qPCR was used to assess the lnc-NEAT1 expression in cells. Firstly, total RNA was extracted from cells using TRIzol solution (Invitrogen, USA); Secondly, RNA was reversely transcribed using PrimeScript RT reagent (Takara, Japan); Thirdly, qPCR was conducted using the One Step SYBR PrimeScript RT-PCR Kit (Takara, Japan). Besides, GAPDH was applied as internal reference in qPCR assay, and lnc-NEAT1 expression was calculated by 2^-△△Ct^ formula. Primers used for qPCR were presented in Table 1.

**Table 1:** Primers in qPCR

Gene	Forward Primer	Reverse Primer
NEAT1	5′ GCCAGTGTGAGTCCTAGCATTG 3′	5′ ACTTCCTCCTCCTAAGCCTCTG 3′
miR-193a	5′ ACACTCCAGCTGGGACTGGGACTTTGTAGGCCA 3′	5′ TGTCGTGGAGTCGGCAATTC 3’
miR-182	5′ ACACTCCAGCTGGGTTTGGCAATGGTAGAACT 3’	5′ TGTCGTGGAGTCGGCAATTC 3’
miR-141	5′ ACACTCCAGCTGGGTAACACTGTCTGGTAAAG 3’	5′ TGTCGTGGAGTCGGCAATTC 3’
U6	5′ CGCTTCGGCAGCACATATACTA 3’	5′ ATGGAACGCTTCACGAATTTGC 3’
GAPDH	5′ GAGTCCACTGGCGTCTTCAC 3’	5′ ATCTTGAGGCTGTTGTCATACTTCT 3’

### CCK8

Cell proliferation ability was assessed by Cell Counting Kit-8 (Dojindo, Japan) at 0 h, 24 h, 48 h and 72 h following the instructions of manufacture. Cells were washed by PBS, and then 10 μL CCK-8 and 90 μL serum free medium were added. After incubation for 2 h under the condition of 95% air plus 5% CO2 at 37 °C, cell proliferation rate was assessed according to the optical density (OD) value (450 nm) detected by Microplate reader (BioTek, USA). Cell Counting Kit-8 (Dojindo, Japan).

### Av/pi

Cell apoptosis rate was assessed by AV/PI assay, and Dead Cell Apoptosis Kit with Annexin V Alexa Fluor™ 488 & Propidium Iodide (Invitrogen, USA) was used. Firstly, 0.25% Tyrisin was used to trypsinize the cells, followed by the termination with serum free medium. Secondly, centrifugation (1800 rpm, 3 min) was performed, and 100 μL suspension was prepared after the supernatant was discarded. Thirdly, 2 μL AV was added in darkness with incubation for 15 mins, and then 1 μL PI was added to cells. Cell apoptosis rate was evaluated by flow cytometry (FCM) (Beckman, USA).

### Statistics

SPSS 19.0 Software (IBM, USA) and GraphPad 6.01 Software (GraphPad, USA) were applied for statistics and graphs in this study. Data were presented as mean ± standard deviation. Comparison among groups was detected by One-Way ANOVA test followed by multiple comparisons test (Dunnett-t test), and comparison between two groups was detected by t test. *P* < 0.05 was considered significant.

## Results

### Validation of myocardial I/R injury model establishment

**Fig. 1:**
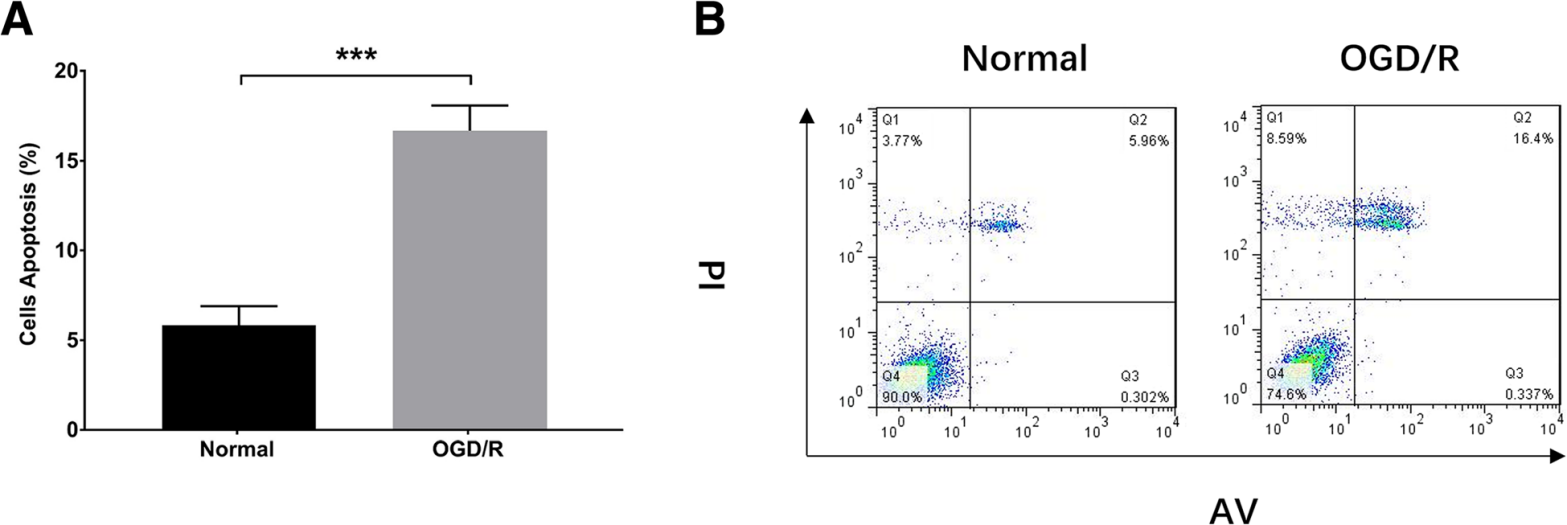
Cell apoptosis rate in OGD/R group and normal group. Cell apoptosis rate was elevated in OGD/R group compared to normal group (**a**-**b**). Comparison between two groups was assessed by t test. OGD/R, oxygen and glucose deprivation followed by reperfusion. *P* < 0.05 was considered significant. ***P < 0.001

**Fig. 2:**
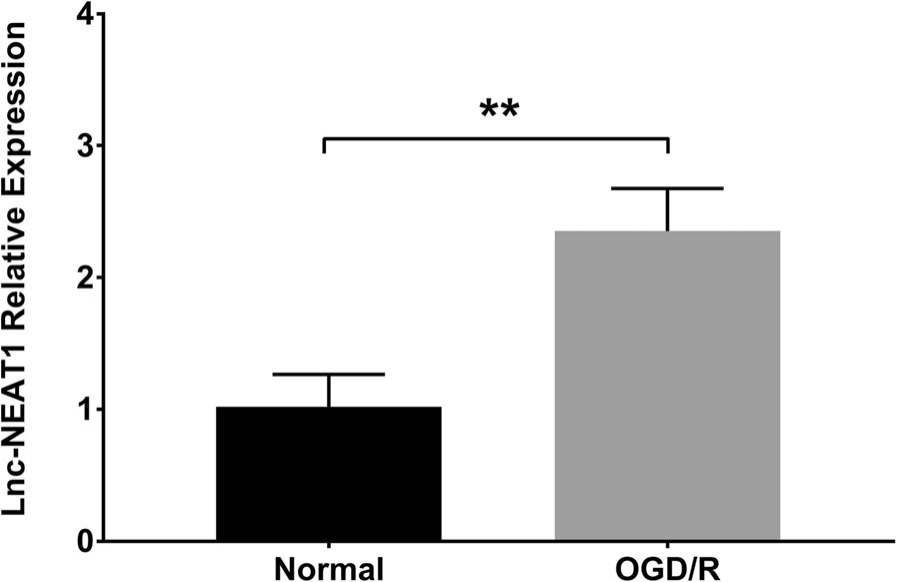
Lnc-NEAT1 expression in OGD/R group and normal group. Compared with normal group, lnc-NEAT1 expression was raised in OGD/R group. OGD/R, oxygen and glucose deprivation followed by reperfusion. P < 0.05 was considered significant. **p < 0.01

Cell apoptosis rate was remarkably higher in OGD/R group compared to normal group (*P* < 0.001), suggesting the successful establishment of myocardial I/R injury model (Fig. 1a-b). Moreover, qPCR was performed to assess the lnc-NEAT1 expression between myocardial I/R injury model and normal myocardial cells, which displayed that lnc-NEAT1 relative expression was elevated in OGD/R group compared to normal group (Fig. 2) (*P* < 0.01).

### Effect of lnc-NEAT1 downregulation on myocardial I/R injury model

**Fig. 3:**
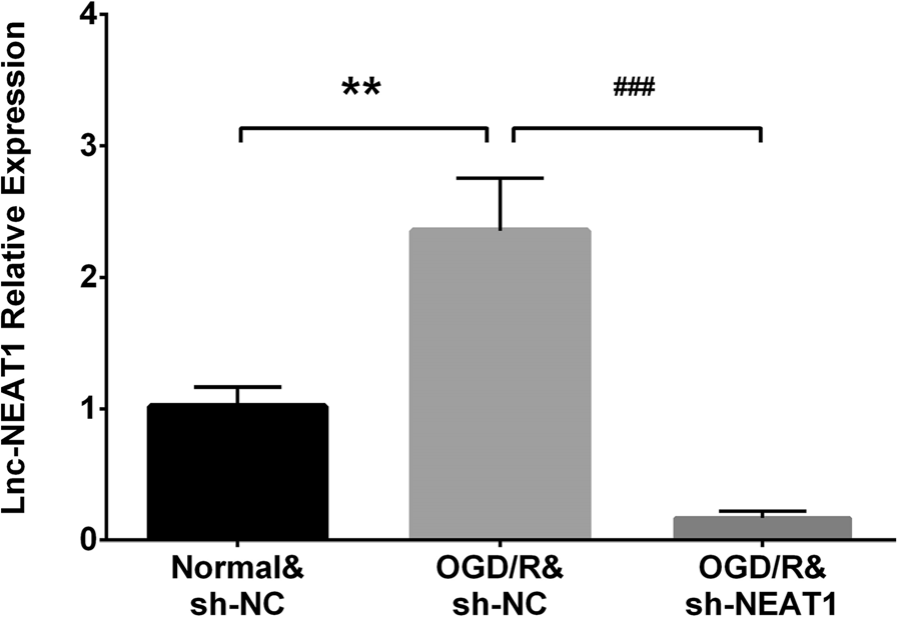
Lnc-NEAT1 expression in OGD/R&sh-NC group, Normal&sh-NC group, OGD/R&sh-NEAT1 group and OGD/R&sh-NC group. Compared to normal&sh-NC group, lnc-NEAT1 expression in OGD/R&sh-NC group was higher, while it was decreased in OGD/R&sh-NC group compared to OGD/R&sh-NC group. Lnc-NEAT1, long non-coding RNA nuclear enriched abundant transcript 1. OGD/R, oxygen and glucose deprivation followed by reperfusion. *p* < 0.05 was considered significant. **p < 0.01, OGD/R&sh-NC group vs. Normal&sh-NC group; ###*p* < 0.001, OGD/R&sh-NEAT1 group vs. OGD/R&sh-NC group

**Fig. 4:**
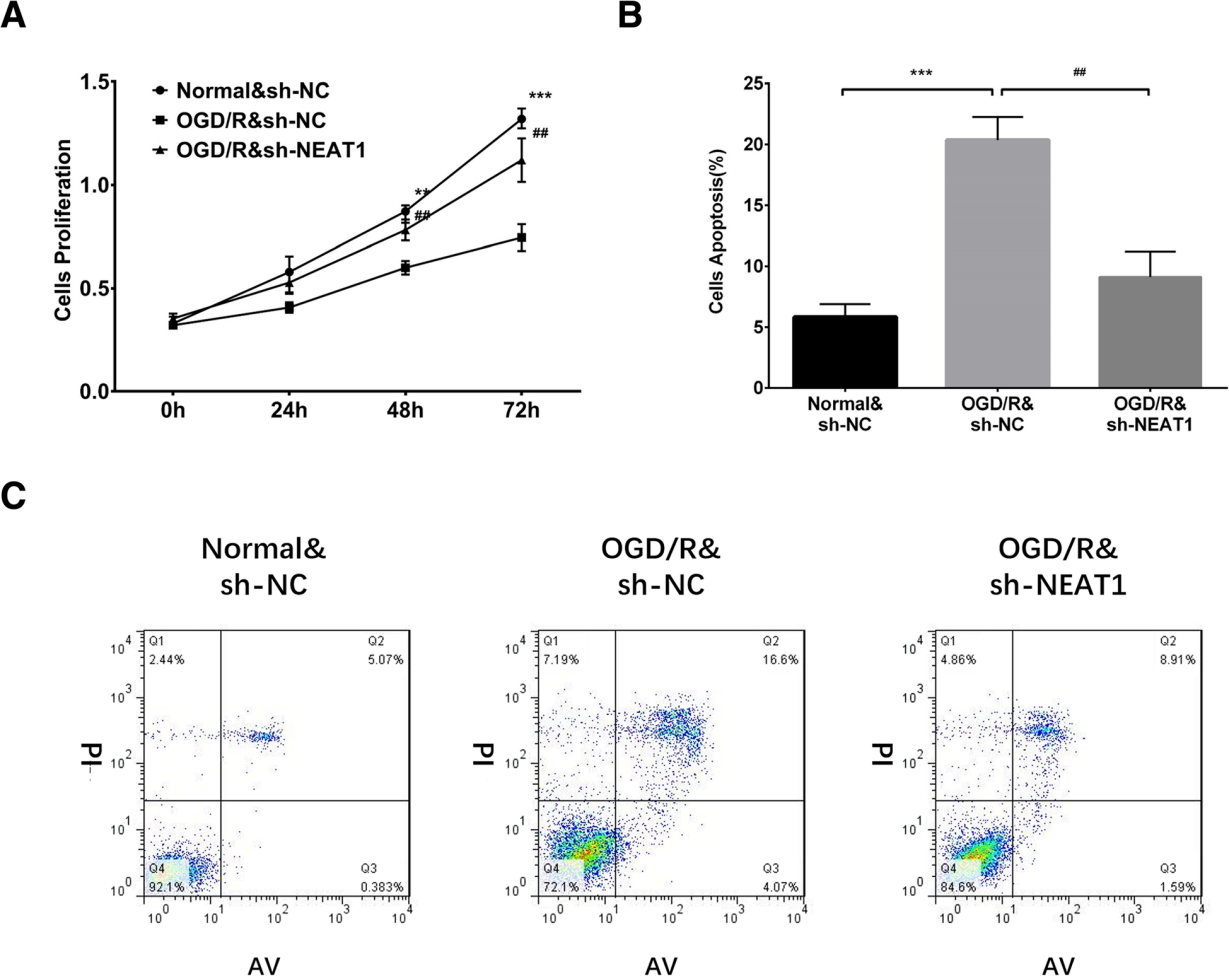
CCK-8 assay and AV/PI assay in lnc-NEAT1 shRNA transfected I/R injury H9c2 cells. Compared to normal&sh-NC group, cell proliferation was reduced in OGD/R&sh-NC group at 48 h and 72 h but increased in OGD/R&sh-NEAT1 group at 48 h and 72 h compared to OGD/R&sh-NC group (**a**). Cell apoptosis rate was raised in OGD/R&sh-NC group compared to normal&sh-NC group at 72 h but decreased in OGC/R&sh-NEAT1 group compared to OGD/R&sh-NC group (**b**-**c**). Lnc-NEAT1, long non-coding RNA nuclear enriched abundant transcript 1. OGD/R, oxygen and glucose deprivation followed by reperfusion. *p* < 0.05 was considered significant. ***p < 0.001, OGD/R&sh-NC group vs. Normal&sh-NC group; **p < 0.01, OGD/R&sh-NC group vs. Normal&sh-NC group; ##*p* < 0.01, OGD/R&sh-NEAT1 group vs. OGD/R&sh-NC group

Lnc-NEAT1 expression was greatly lower in OGD/R&sh-NEAT1 group compared to OGD/R&sh-NC group, indicating the successful transfection of lnc-NEAT1 shRNA plasmids (Fig. 3) (*P* < 0.001). Cell proliferation rate was increased in OGD/R&sh-NEAT1 group at 48 h (*P* < 0.01) and 72 h (P < 0.01) compared to OGD/R&sh-NC group (Fig. 4a). For cell apoptosis rate, it was lower in OGC/R&sh-NEAT1 group compared to OGD/R&sh-NC group (Fig. 4b-c) (*P* < 0.01). These results suggested that downregulation of lnc-NEAT1 enhanced cell proliferation and repressed cell apoptosis in I/R injury H9c2 cells.

### Expressions of candidate target miRNAs of lnc-NEAT1

**Fig. 5:**
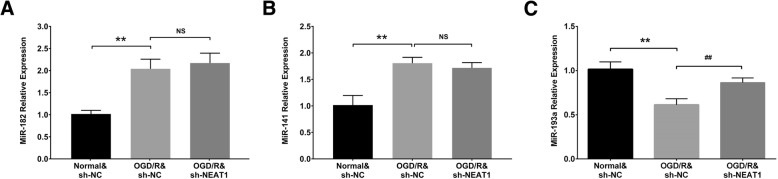
Prediction and detection of target miRNAs of lnc-NEAT1. Both miR-182 expression and miR-141 expression were elevated in OGD/R&sh-NC group compared to normal&sh-NC group at 24 h, while no difference of miR-182 expression or miR-141 expression was found between OGC/R&sh-NEAT1 group and OGD/R&sh-NC group (**a**-**b**). Furthermore, miR-193a expression was reduced at 24 h in OGD/R&sh-NC group compared to normal&sh-NC group but raised in OGC/R&sh-NEAT1 group compared to OGD/R&sh-NC group at 24 h. Lnc-NEAT1, long non-coding RNA nuclear enriched abundant transcript 1. OGD/R, oxygen and glucose deprivation followed by reperfusion. *P* < 0.05 was considered significant. **, *p* < 0.01, OGD/R&sh-NC group vs. Normal&sh-NC group; ##, *p* < 0.01, OGD/R&sh-NEAT1 group vs. OGD/R&sh-NC group; NS, no significant difference

In order to further explore the detailed mechanism of lnc-NEAT1 in myocardial I/R injury, expressions of candidate target miRNAs of lnc-NEAT1 were evaluated (Fig. 5). And we observed that miR-193a was downregulated while miR-182 and miR-141 were upregulated in OGD/R&sh-NC group compared to normal&sh-NC group, indicating that these miRNAs play a role in the myocardial I/R injury. Most importantly, miR-193a expression was increased but miR-182 and miR-141 expressions were undifferentiated in OGC/R&sh-NEAT1 group compared with OGD/R&sh-NC group, suggesting that only miR-193a was targeted by lnc-NEAT1 in I/R injury H9c2 cells.

### Rescue experiments

**Fig. 6:**
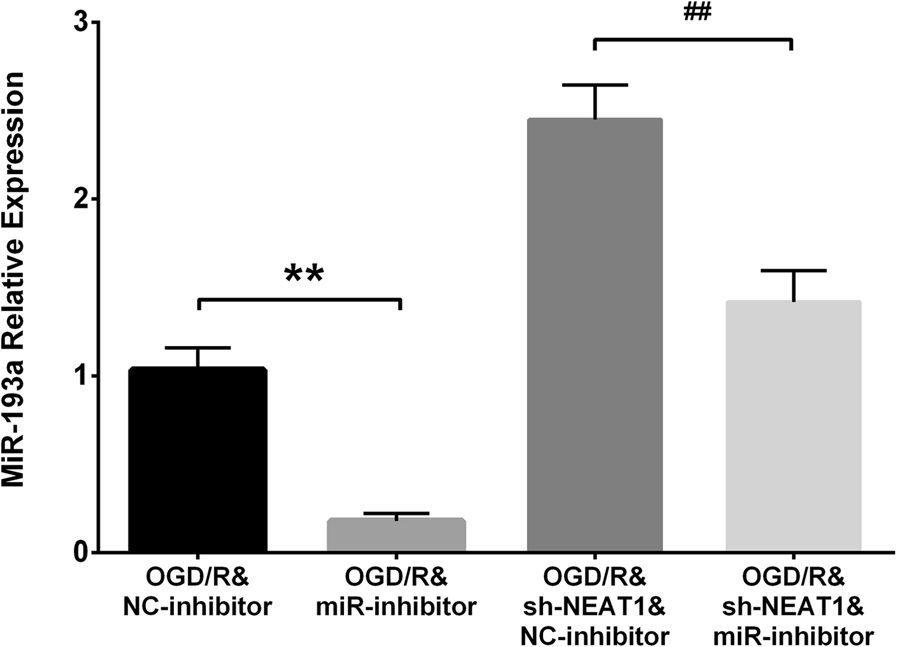
Measurement of miR-193a after transfected with NEAT1 shRNA and miR-193a inhibitor plasmids. Compared to OGD/R&NC-inhibitor group, miR-193a expression was lower in OGD/R&miR-inhibitor group, whereas it was decreased in sh-NEAT1&miR-inhibitor group compared to sh-NEAT1&NC-inhibitor group at 24 h. Lnc-NEAT1, long non-coding RNA nuclear enriched abundant transcript 1. OGD/R, oxygen and glucose deprivation followed by reperfusion. *p* < 0.05 was considered significant. **p < 0.01, OGD/R&miR-inhibitor group vs. OGD/R&NC-inhibitor group; ##*p* < 0.01, OGD/R&sh-NEAT1&miR-inhibitor group vs. OGD/R&sh-NEAT1&NC-inhibitor group

**Fig. 7:**
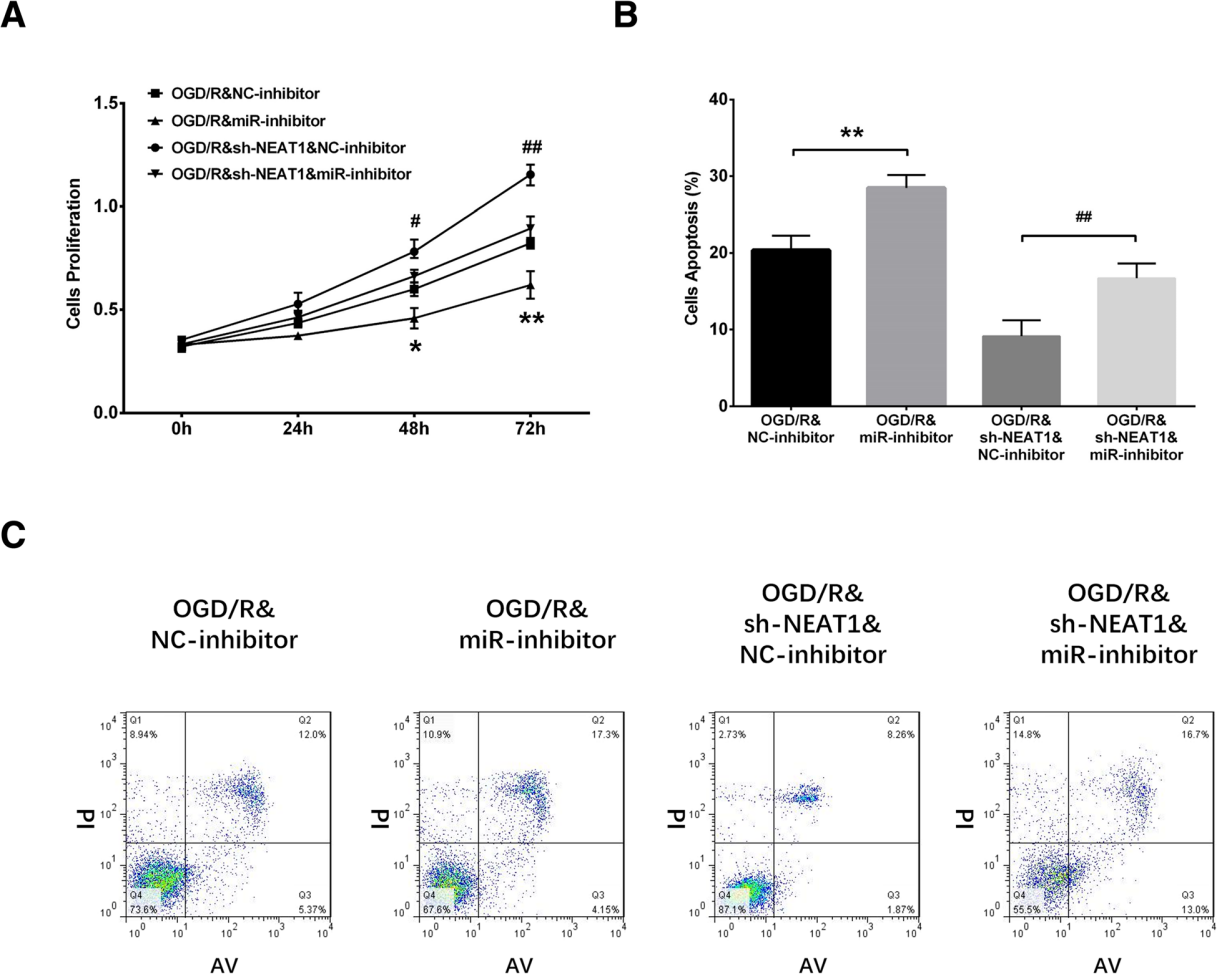
Rescue experiments. Cell proliferation rate was decreased in in OGD/R&miR-inhibitor group compared to OGD/R&NC-inhibitor group at 48 h and 72 h, but it was reduced in OGD/R&sh-NEAT1&miR-inhibitor group compared to OGD/R&sh-NEAT1&NC-inhibitor group at 48 h and 72 h. In addition, cell apoptosis rate was elevated in OGD/R&miR-inhibitor group compared to OGD/R&NC-inhibitor group at 72 h and increased in OGD/R&sh-NEAT1&miR-inhibitor group compared to OGD/R&sh-NEAT1&NC-inhibitor group at 72 h. Lnc-NEAT1, long non-coding RNA nuclear enriched abundant transcript 1. OGD/R, oxygen and glucose deprivation followed by reperfusion. *p* < 0.05 was considered significant. **p < 0.01, OGD/R&miR-inhibitor group vs. OGD/R&NC-inhibitor group; *p < 0.05, OGD/R&miR-inhibitor group vs. OGD/R&NC-inhibitor group; ##*p <* 0.01, OGD/R&sh-NEAT1&miR-inhibitor group vs. OGD/R&sh-NEAT1&NC-inhibitor group; #*p* < 0.05, OGD/R&sh-NEAT1&miR-inhibitor group vs. OGD/R&sh-NEAT1&NC-inhibitor group

In order to identify whether lnc-NEAT1 regulates H9c2 cells functions through targeting miR-193a, rescue experiment was performed, which disclosed that miR-193a expression was reduced in OGD/R&miR-inhibitor group compared to OGD/R&NC-inhibitor group (*P* < 0.01), while it was lower in sh-NEAT1&miR-inhibitor group compared to sh-NEAT1&NC-inhibitor group at 24 h, indicating the successful transfections of miR-193a inhibitor plasmids in I/R injury H9c2 cells (Fig. 6) (*P* < 0.01). In the followed CCK8 assay, cell proliferation rate was reduced in OGD/R&miR-inhibitor group compared to OGD/R&NC-inhibitor group at 48 h (*P* < 0.05) and 72 h (*P* < 0.01), and it decreased in OGD/R&sh-NEAT1&miR-inhibitor group compared to OGD/R&sh-NEAT1&NC-inhibitor group at 48 h (*P* < 0.05) and 72 h (*P* < 0.01) (Fig. 7a), suggesting that downregulation of lnc-NEAT1 promoted cell proliferation via targeting miR-193a in I/R injury H9c2 cells. Furthermore, cell apoptosis rate detected by AV/PI assay was increased in OGD/R&miR-inhibitor group compared to OGD/R&NC-inhibitor group at 72 h (*P* < 0.01), and it was elevated in OGD/R&sh-NEAT1&miR-inhibitor group compared to OGD/R&sh-NEAT1&NC-inhibitor group at 72 h (Fig. 7b-c) (*P* < 0.01), suggesting that downregulation of lnc-NEAT1 inhibited cell apoptosis through targeting miR-193a in I/R injury H9c2 cells.

## Discussion

In this study, we found that: (1) lnc-NEAT1 expression was higher in I/R injury H9c2 cells compared to normal H9c2 cells, and down-regulation of lnc-NEAT1 promoted cell proliferation and inhibited apoptosis in I/R injury H9c2 cells; (2) rescue experiment revealed that down-regulation of lnc-NEAT1 enhanced cell proliferation and repressed cell apoptosis via targeting miR-193a in I/R injury H9c2 cells.

LncRNAs acts as new modulators involving in physiological processes to maintain cellular and tissue homeostasis, and parts of their dysregulated expressions devote into the onset and progression of multiple pathological conditions, including the pathophysiology of myocardial I/R injury [18]. For instance, lncRNA Reprogramming (ROR) reduces cells viability and promotes cell apoptosis through improving p38 phosphorylation, inhibiting p38/MAPK and rescuing lncRNA-ROR-induced cell injury in H9c2 cells [18]. Besides, lncRNA urothelial carcinoma-associated 1 (UCA1) contributes to cardiomyocyte apoptosis via suppressing p27 level in rat cardiac ischemia reperfusion injury [19]. Another study displays that downregulation of lncRNA-MALAT1 enhances cardiomyocyte apoptosis by targeting miR-145/Bnip3 pathway. Thus, these evidences reveal that lncRNAs involve into the pathological processes of the myocardial I/R injury.

For lnc-NEAT1, it has been reported to be abnormally expressed in various diseases, and it could affect cell proliferation, cells migration and cells invasion through regulating multiple genes or pathways such as Ras-mitogen-activated protein kinase (MAPK), Wnt/β-catenin pathway and miR-377-3p-E2F3 pathways [9, 20]. For example, a study conducted by Sun et al. displays that lnc-NEAT1 promotes cell proliferation in non-small cell lung cancer, and another study discloses that lnc-NEAT1 enhances cell proliferation, invasion and migration in endometrial endometrioid adenocarcinoma cells [20, 21]. Furthermore, a review reveals that neutrophils play important role in I/R injury due to their proximity at endothelium and other inflammatory cells at the vascular interface [22]. Once neutrophil adhesion is established, neutrophils infiltrate the ischemic tissues, and neutrophil infiltration into the infarcted area indicates the generation of reactive oxygen species (ROS) and proteolytic enzymes that injure the surrounding cardiomyocytes. Additionally, macrophages show both inhibitory actions by secreting mediators suppressing inflammation and pro-resolving actions in order to remove inflammatory leucocytes during postinfarction inflammation [22]. For lnc-NEAT1, one study shows that lnc-NEAT1 presents higher expression in neutrophils compared to other immune cells and lnc-NEAT1 may derive from metastatic tissues and neutrophils, meanwhile, lnc-NEAT1 promotes ox-LDL-induced inflammation and oxidative stress in macrophages [23, 24]. Although these previous studies reveal that lnc-NEAT1 serves as an oncogene in various cancers through promoting cell proliferation and inhibiting cell apoptosis, moreover, lnc-NEAT1 may present influences in neutrophils and macrophages, which are inflammatory cells involved in the myocardial I/R injury, little is known about the effect of lnc-NEAT1 on cells activities in cardiovascular diseases, particularly myocardial I/R injury. In this study, we detected lnc-NEAT1 expression in myocardial I/R injury cells as well as normal myocardial cells, and we found that lnc-NEAT1 expression was elevated in myocardial I/R injury cells compared to normal cells, moreover, downregulation of lnc-NEAT1 increased myocardial I/R injury cell proliferation but reduced cell apoptosis, indicating that lnc-NEAT1 might play an unfavorable role in protecting against myocardial ischemia, and the downregulation of lnc-NEAT1 might shed a light to the treatment of myocardial ischemia due to its promotion of myocardial cell proliferation and inhibition of cell apoptosis. In addition, only the rat cardiac muscle cell line H9c2 was available for this study due to the scarcity in diversity of myocardial cells in China, which resulted in the lack of verification in other cell lines, and similar limitations could be observed in some previous studies that focus on myocardial ischemia/reperfusion (I/R) injury [25, 26].

MicroRNA-193a (miR-193a), which is embedded in a CpG island, has been identified as a tumor suppressive miRNA in several cancers such as oral carcinoma, lung cancer and colorectal cancer [27–33]. Apart from cancers, it is disclosed that miR-193a is downregulated in intestinal inflammation in colitis, and upregulation of miR-193a promotes anti-inflammatory activities via targeting apoptotic pathways death receptor-6 (DR6) [34]. Hence, these previous evidences reveal that miR-193a influence cells activities in diverse diseases, thereby devoting to these disease developments and progressions, whereas few evidences of miR-193a in affecting I/R injury myocardial cells have been reported. In this present study, we found that downregulation of lnc-NEAT1 promoted cell proliferation and repressed apoptosis by targeting miR-193a, furthermore, we conducted rescue experiments, which revealed that down-regulating lnc-NEAT1 affected cell proliferation and cell apoptosis through regulating miR-193a in I/R injury H9c2 cells, and these results might provide indications for further exploring mechanisms of myocardial I/R injury. Considering the over-expression of lnc-NEAT1 in myocardial I/R injury cells, and the promotion of myocardial cell proliferation as well as inhibition of cell apoptosis by lnc-NEAT1 downregulation, lnc-NEAT1 had the potential to applied in better stratification of patients suffering from myocardial I/R injury and be a novel target for treatment of myocardial I/R injury. Further study is needed to verify our findings as well as investigate the role of lnc-NEAT1 in myocardial I/R injury with clinical practices.

## Conclusions

lnc-NEAT1 is overexpressed in myocardial I/R injury cells compared to normal myocardial cells, and downregulation of lnc-NEAT1 enhances cell proliferation while inhibits cell apoptosis through targeting miR-193a in I/R injury H9c2 cells.

## Data Availability

All relevant data is presented in the manuscript and supporting material.

## Abbreviations

DMEM: Dulbecco’s Modified Eagle’s Medium
DR6: death receptor-6
FBS: Fetal Bovine Serum
FCM: flow cytometry
I/R: ischemia/reperfusion
lnc-NEAT1: LncRNA nuclear enriched abundant transcript 1
lncRNAs: long non-coding RNAs
MAPK: Ras-mitogen-activated protein kinase
miR-193a: MicroRNA-193a
OGD/R: oxygen and glucose deprivation followed by reperfusion
ROR: Reprogramming
UCA1: urothelial carcinoma-associated 1
